# An Economical High-Throughput Protocol for Multidimensional Fractionation of Proteins

**DOI:** 10.1155/2012/735132

**Published:** 2012-09-12

**Authors:** David John Tooth, Varun Gopala Krishna, Robert Layfield

**Affiliations:** School of Biomedical Sciences, University of Nottingham Medical School, Queen's Medical Centre, Nottingham NG7 2UH, UK

## Abstract

A sequential protocol of multidimensional fractionation was optimised to enable the comparative profiling of fractions of proteomes from cultured human cells. Differential detergent fractionation was employed as a first step to obtain fractions enriched for cytosolic, membrane/organelle, nuclear, and cytoskeletal proteins. Following buffer exchange using gel-permeation chromatography, cytosolic proteins were further fractionated by 2-dimensional chromatography employing anion-exchange followed by reversed-phase steps. Chromatographic fractions were shown to be readily compatible with 1- and 2-dimensional gel electrophoresis or with direct analysis by mass spectrometry using linear-MALDI-TOF-MS. Precision of extraction was confirmed by reproducible SDS-PAGE profiles, MALDI-TOF-MS spectra, and quantitation of trypsinolytic peptides using LC-MS/MS (MRM) analyses. Solid phases were immobilised in disposable cartridges and mobile-phase flow was achieved using a combination of centrifugation and vacuum pumping. These approaches yielded parallel sample handling which was limited only by the capacities of the employed devices and which enabled both high-throughput and experimentally precise procedures, as demonstrated by the processing of experimental replicates. Protocols were employed at 10 mg scale of extracted cell protein, but these approaches would be directly applicable to both smaller and larger quantities merely by adjusting the employed solid- and mobile-phase volumes. Additional potential applications of the fractionation protocol are briefly described.

## 1. Introduction

Protein identification and quantitation are major steps towards full characterization of a proteome. Many proteomic projects classically employ 2-dimensional gel electrophoresis (2DE) and are limited by both the precision of the technique and by well-documented limitations in pI and molecular size constraints [1]. Proteome fractionation is desirable in potentially yielding reduced complexity and increased dynamic range and there have been numerous approaches developed including affinity-depletion [2] and immune depletion of major components [3], liquid isoelectric focussing (IEF) [4], GelC-MS [5], and multidimensional column liquid chromatographic (MDLC) protocols [6].

Differential detergent fractionation (DDF) has long been proposed a suitably robust alternative to more challenging and costly differential ultracentrifugation approaches [7] and indeed its use was recently commercialised [8].

For several decades, liquid chromatography has been a powerful tool for separating proteins, peptides, and other molecules in complex mixtures [9]. Users employ exclusively pumped systems, disadvantages of which are inherently low throughput and no opportunity for parallel processing; the applications of such approaches have been reviewed [10–12]. Two-dimensional systems were also commercialised and their uses have been cited in several proteomics applications [13, 14]. MDLC has been commonly employed more recently for increased separation of complex peptide mixtures to enable increased mass-spectrometer experimental time and so maximised protein structural analysis, either incorporating offline MDLC [15] automated online [16] or using biphasic columns in MuDPIT approaches [17]. Potential disadvantages of these latter peptide MDLC experiments are the disparate nature of peptide analyses and the potential transparency of some posttranslational processing which may be overcome by alternatively using or combining prior protein fractionation.

Gel permeation chromatography (GPC) separates proteins and smaller components on the basis of molecular weight and three-dimensional shape [18]. Components move through a bed of porous beads, with smaller molecules diffusing further into pores and moving more slowly, whilst larger molecules enter less or not at all, so passing through more quickly. GPC has been used analytically or for buffer exchange in preparative work flows.

Ion-exchange chromatography separates proteins based on differences between pI and net charge [9]. Proteins must have a charge opposite that of the functional group attached to the resin in order to bind. For example, at pH 10, proteins with pI below approximately 9 have a net negative charge and bind to anion exchangers which contain positively charged functional groups. Because this interaction is ionic, binding must take place under low ionic conditions and elution is achieved either by increasing the ionic strength or decreasing the pH of the mobile phase. Mobile phases typically employed in ion exchange are well suited to direct orthogonal second-dimensional separation using reversed-phase chromatography and there are numerous published examples [6, 13, 14].

Reversed-phase chromatography has been and is commonly employed as the final chromatographic stage in proteomics workflows due to the volatile nature of the mobile phase which makes it compatible with both on- and off-line mass spectrometric analyses. Example potential applications include analyses of tissue specimens using MALDI-TOF-MS in studies to design discriminatory disease biomarkers [19] and quantitative proteomic studies employing LC-MS/MS methods such as multireaction monitoring (MRM) which has been recently reviewed [20]. Reversed-phase fractions are suitably stable samples for storage, at least in the short term, and may be readily dried or lyophilised using vacuum devices for longer-term storage or additionally to be made compatible with electrophoretic methods. Another approach to which this protocol, using all described dimensions, could potentially be applied is GeLCMS whereby regions of SDS-PAGE profiles are proteolysed and subsequently analysed using mass-spectrometry [21].

Here, using human cell extracts as an exemplar we demonstrate how a number of these fractionation approaches can be combined to afford the multidimensional separation of protein mixtures in an economical and high-throughput protocol, which is broadly applicable to a range of experimental scales.

## 2. Methods

### 2.1. Cell Harvesting and Protein Extraction

Samples of cultured human Neuro-2A cells were harvested in triplicate. Each set of triplicates represented cells in which wild-type or mutant malin or laforin proteins were transiently overexpressed [22] although for the purposes of this paper the malin/laforin phenotype is irrelevant. Similar to 5 × 10^7^ cells, sufficient to yield approximately 10 mg of total protein were removed from culture dishes using trypsin and then pelleted by centrifugation. Following washing with phosphate-buffered saline (PBS) twice, pellets were rapidly frozen using liquid nitrogen and stored at −70°C.

Differential detergent fractionation (DDF) was performed with optimised modifications to published methods [7, 8]. Cells were initially washed with 1-cell-pellet-volume of PBS containing 1 : 100 protease inhibitor cocktail (Sigma; P8340) and 5 *μ*g·mL^−1^ proteasome inhibitor MG132. Pellets were resuspended with 2.5 volumes freshly prepared 0.01% (w/v) digitonin in extraction buffer (5 mM EDTA, 1 : 200 phosphatase inhibitor [Roche; PhosStop] 1 : 100 protease inhibitor and 5 *μ*g·mL^−1^ MG132 in 50 mM HEPES, 150 mM sodium chloride pH 7.4) and following a brief agitation incubated on ice for 10 minutes. Supernatants enriched in cytosolic proteins were recovered and the extraction repeated. Membrane/organelle protein-enriched fractions were obtained in a similar procedure but using 1% (w/v) IGEPAL in the same extraction buffer. Nuclear extracts were prepared using 1.5 volumes of 0.25% (w/v) deoxycholate, 0.1% (w/v) SDS, and 500 units·mL^−1^ benzonase, in extraction buffer. Finally, cytoskeletal protein-enriched fractions were obtained using 1.5 volumes of 0.25% (w/v) deoxycholate, 1.0% (w/v) SDS in extraction buffer. All protein quantitation was performed using a commercially available (Sigma) Bicinchoninic acid (BCA) kit, which was determined to demonstrate similar to 100–120% accuracies using the described DDF and anion-exchange buffers (data not shown).

### 2.2. Anion-Exchange Fractionation

Buffer exchange was firstly performed upon cytosolic protein extracts by (centrifugally) passing through GPC columns prepared using 1 g resin (GE Healthcare, G-25 Sepharose) per mL of sample, preequilibrated with (10 mL per gramme of resin) chromatography load buffer (8 M urea, 20 mM Tris, pH 10.0). A strong anion-exchange resin was selected (BioRad; UNOsphere-Q) which has a monoquaternary amine functional group with 120 *μ*m bead diameter and protein binding capacities in excess of 100 mg·mL^−1^. Anion-exchange columns were prepared using 0.2 mL resin per mg cytosolic proteins, preconditioned with 10 resin volumes of load buffer; extracts were sequentially passed 3 times and retained components were washed thrice with 3 resin volumes of load buffer. Selective elution was achieved using a sequential application of 0.15, 0.25, 0.40, and 1.0 M potassium chloride (in load buffer) and all flow-through, wash and eluate fractions were retained. Disposable columns were prepared using fritted polypropylene extraction tubes (Supelco), although centrifugal filtration microtubes or filter-microplates would be suitable for smaller-scale or higher-throughput applications, respectively. Chromatographic flow was achieved using centrifugation (1000 ×g) for typically 1 minute or until columns were emptied.

### 2.3. Reversed-Phase Fractionation

Polymeric large-pore reversed-phase SPE cartridges (IST ISOLUTE, PDVB, 1000 Å, 25 mg) were selected and had previously been assessed to display broad binding selectivities with capacities of similar to 0.1 mg·mg^−1^ (data not shown). Solid phase was initially “wetted” with 1 mL (per 25 mg) 70% (v/v) acetonitrile, 0.1% (v/v) trifluoroacetic acid (TFA) in water and preconditioned with 1 mL 0.1% (v/v) TFA in water. Anion-exchange fractions were loaded, washed with 0.1% (v/v) TFA in water and sequentially eluted with 1 mL of 30, 35, 40, 43, 45, 48, 50, and 90% (v/v) acetonitrile, 0.1% (v/v) TFA in water. Chromatographic flow was achieved using vacuum pumping and the target mobile-phase flow-rate was 1 mL·min^−1^.

### 2.4. Protein Electrophoresis

DDF extracts were loaded directly on to SDS-PAGE gels (Invitrogen, 4–12% Bis-Tris, MES) following dilution in sample buffer. MDLC fractions were first dried to residue using vacuum centrifugation then resolubilised in sample buffer. Protein components were visualised using Coomassie Blue G-250 [23] chemical staining. 2DE was performed using IPG-strip IEF gels (BioRad, 11 cm, 3–10) with dried samples resuspended in 8 M urea, 2% (w/v) CHAPS, 50 mM DTT and subsequent IEF for in excess of 30,000 Vh. Second-dimensional SDS-PAGE was as described previously and proteins were silver-stained using a standard protocol [24].

### 2.5. MALDI-TOF Mass-Spectrometric Analysis

Mass spectrometric profiling of MDLC fractions, using linear-MALDI-TOF-MS, was performed by direct cospotting of reversed-phase SPE eluates with saturated solutions of sinapic acid (Fluka) or alpha-cyano-4-hydroxy-cinnamic acid (Sigma-Aldrich) in 50% (v/v) acetonitrile, 0.1% (v/v) TFA in water. A LaserToF TT (SAI Ltd.) operated in positive ion and linear modes was used to acquire spectra over the 1,000–300,000 m/z range and was calibrated against a range of protein standards.

### 2.6. Liquid-Chromatography-Tandem-Mass-Spectrometric Analysis

Trypsinolytic peptides recovered from selected MDLC fractions were analysed by microcapillary-LC-MS/MS using a hybrid Q-TOF instrument (Waters QToF2) equipped with a nanoelectrospray ion-source and controlled using MassLynx 4.0 software. Data-dependent product ion experiments were performed and protein identifications were ascertained using the MS/MS Ion Search program in the MASCOT search engine (http://www.matrixscience.com/). Selected signature peptides identified in MDLC fractions were analysed quantitatively by LC-MS/MS using a triple-quadrupole instrument (Waters Quattro Ultima) using analytical scale HPLC and controlled using MassLynx 4.0 software. MRM experiments were performed using previously identified precursor and product ions.

## 3. Results

### 3.1. Fractionation Achieved Using Differential Detergent Protocol

**Figure 1:**
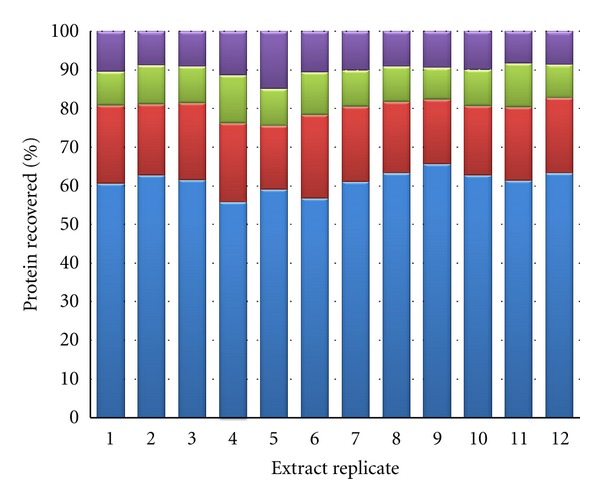
The distribution of extracted proteins in subcellular proteome fractions from twelve samples of cultured human Neuro-2A cells using the described DDF protocol (cellular phenotypes randomised and denoted replicates 1–12). Proteins were quantitated using a BCA method and quantities were normalised as a percentage of total extracted protein. Fractions denoted are cytosolic (blue), membrane/organelle (red), nuclear (green), and cytoskeletal (violet).

**Figure 2:**
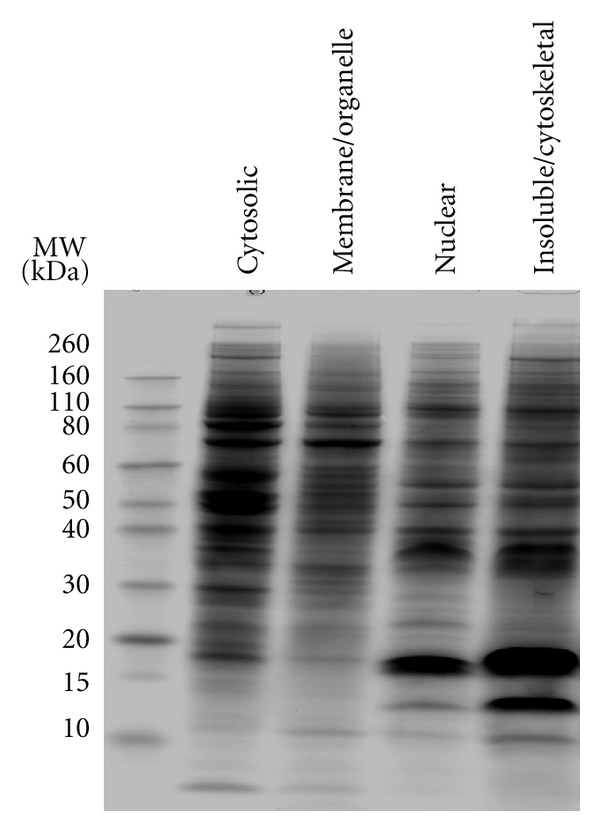
Exemplary analytical SDS-PAGE showing the profiles of proteins in subcellular proteome fractions extracted from a single sample of cultured human Neuro-2A cells, using the described DDF protocol. Similar to 20 *μ*g of protein in each fraction was loaded and subsequently chemically stained using Coomassie Blue G-250. Partial differential fractionation is clearly evident.

Reproducible protein extraction and fractionation was achieved for triplicates of the 4 experimental cell phenotypes using the described DDF protocol, evidenced from comparable percentage protein distribution in the resulting fractions (Figure 1). SDS-PAGE profiling of DDF extracts demonstrated clear differences in protein constituents of the fractions, providing evidence of successful subcellular fractionation (Figure 2).

### 3.2. Anion-Exchange Fractionation of Cytosolic Proteins

**Figure 3:**
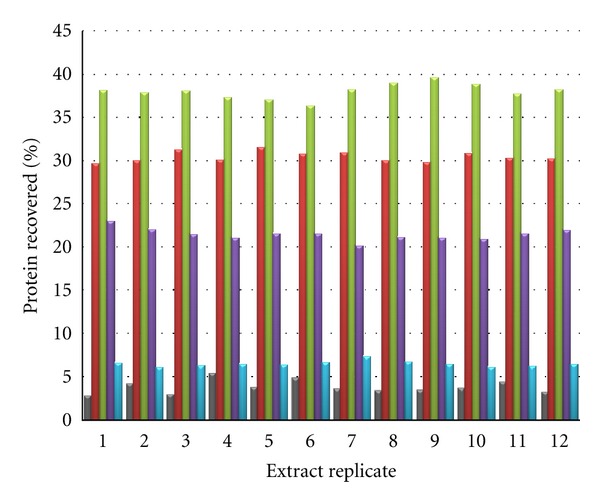
The distribution of cytosolic proteins, extracted from twelve samples of cultured human Neuro-2A cells, in anion-exchange chromatographic fractions. Proteins were quantitated using a BCA method and quantities were normalised as a percentage of total extracted protein. The flow-through (grey), 0.15 M (red), 0.25 M (green), 0.4 M (violet) and 1.0 M (blue) potassium chloride eluate chromatographic fractions display high between sample precision using the anion-exchange protocol described.

Each of the twelve cytosolic protein fractions was further fractionated by anion-exchange chromatography using the described protocol. Less than 5% of protein was unretained and the chromatographed cytosolic proteomes were distributed throughout the four eluted fractions. Again, precise and reproducible protein fractionation was achieved, evidenced from comparable percentage protein distribution in the resulting fractions (Figure 3). In this case, the employed eluent compositions had been optimised empirically but further improved fractionation, by more refined choice of eluent, would clearly be possible so yielding either increased fractionation or more equally distributed mass quantities of proteins as required.

### 3.3. Second-Dimensional Reversed-Phase Separation

**Figure 4:**
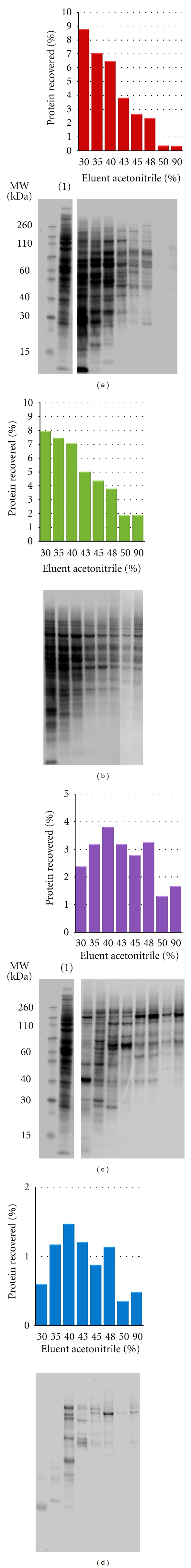
The distribution of cytosolic proteins in reversed-phase chromatographic fractions of 0.15 M (a), 0.25 M (b), 0.4 M (c), and 1.0 M (d) potassium chloride anion-exchange fractions, derived from a single DDF extract of cultured human Neuro-2A cells. Proteins were quantitated using a BCA method and quantities were normalised as a percentage of total extracted protein (top). Samples of all fractions were analysed using SDS-PAGE stained with Coomassie Blue G-250 (bottom) and total cytosolic extract is also shown (1) for comparison. It is evident that proteins from each of the anion-exchange fractions eluted over a broad range of hydrophobicity using the reversed-phase protocol and were therefore further fractionated.

**Figure 5:**
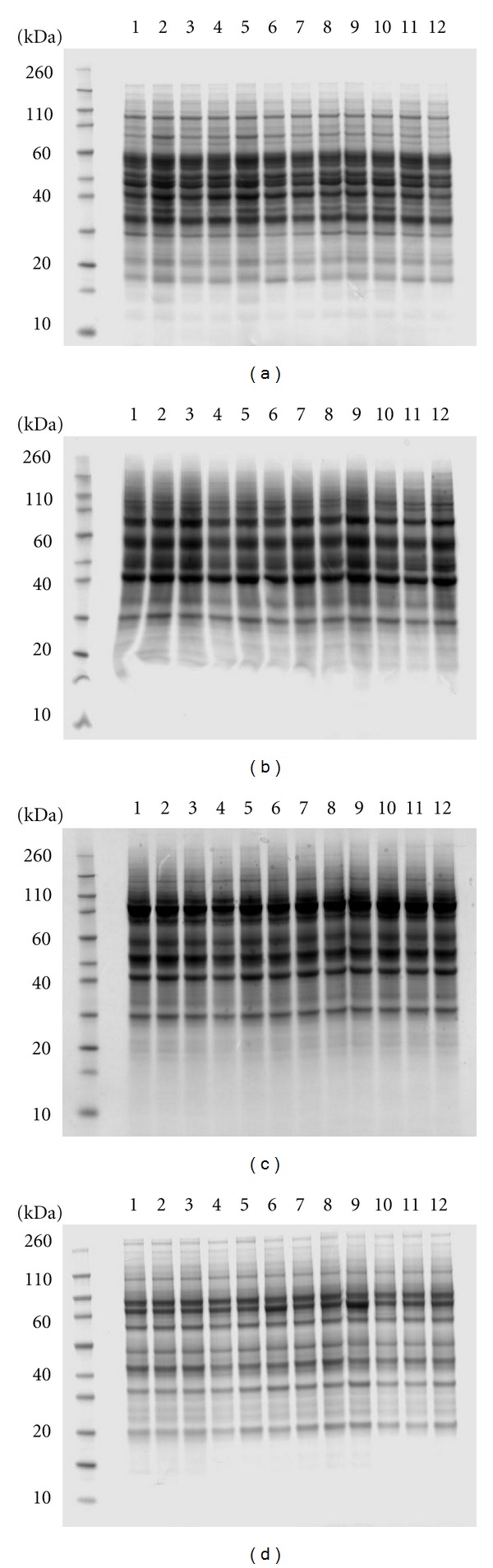
Representative comparative Coomassie Blue G250-stained analytical SDS-PAGE analyses of four MDLC fractions. These are in all cases the 45% (v/v) acetonitrile reversed-phase chromatographic fractions of 0.15 M (a), 0.25 M (b), 0.4 M (c), and 1.0 M (d) potassium chloride anion-exchange fractions, originally derived from cytosolic DDF extracts of twelve samples of cultured human Neuro-2A cells.

**Figure 6:**
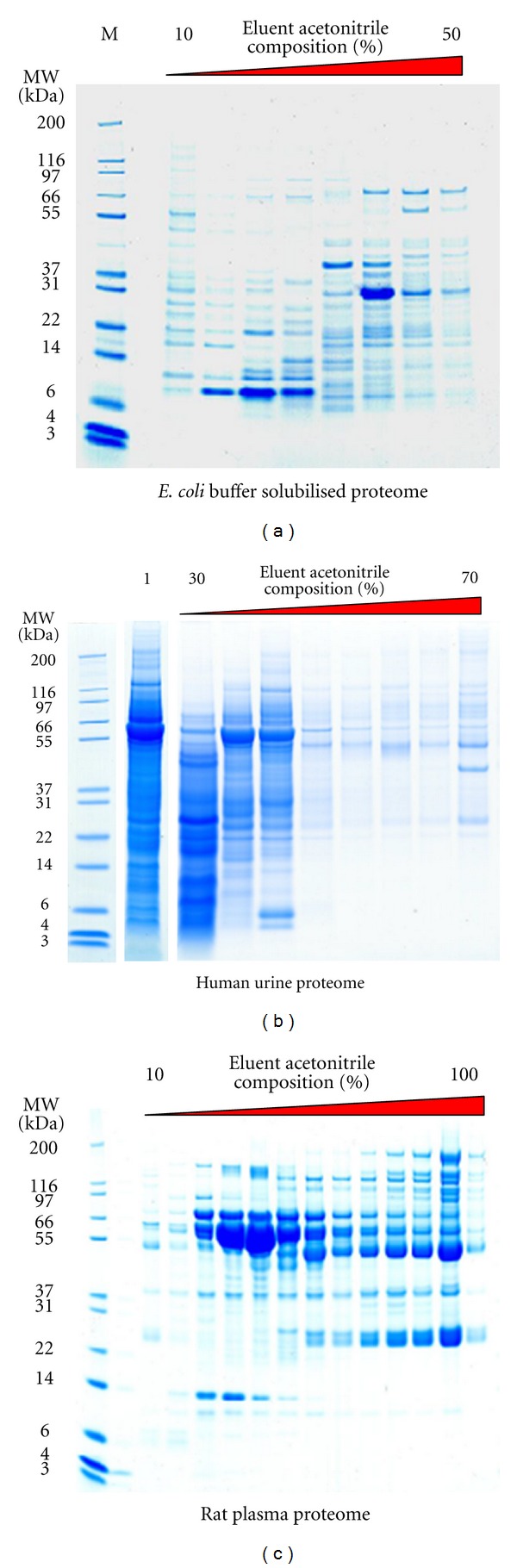
Versatile applicability of reversed-phase SPE alone which enables the concentration, desalting and partial fractionation of complex proteome samples. Coomassie Blue G250-stained analytical SDS-PAGE of reversed-phase SPE fractionated samples equivalent to (a) approximately 50 *μ*g of *E. coli* whole cell lysate, (b) 1 mL of human urine, also unfractionated (1), and (c) 0.01 mL of rat plasma.

Proteome distribution in reversed-phase fractions of exemplary anion-exchange fractions (derived from a single cytosolic protein fraction) is shown in Figures 4(a)–4(d). Again the chosen eluent compositions were optimised empirically; however, very similar performance was achieved when this protocol had been previously applied to a range of alternative proteome samples. Comparative analysis of experimental test cell types was enabled using the methods described here. Examples of analytical SDS-PAGE of various MDLC fractions are shown in Figures 5(a)–5(d). The high precision of the fractionation protocols is apparent (note the reproducibility of the gel band profiles) and the potential for comparative proteome analysis is clearly evident. Figures 6(a)–6(c) show fractionation of plasma, urine, and bacterial cell lysate and highlight that this protocol on its own offers a powerful and broadly applicable fractionation strategy.

### 3.4. Proteome Profiling Using MALDI-TOF-Mass-Spectrometric Analysis

**Figure 7:**
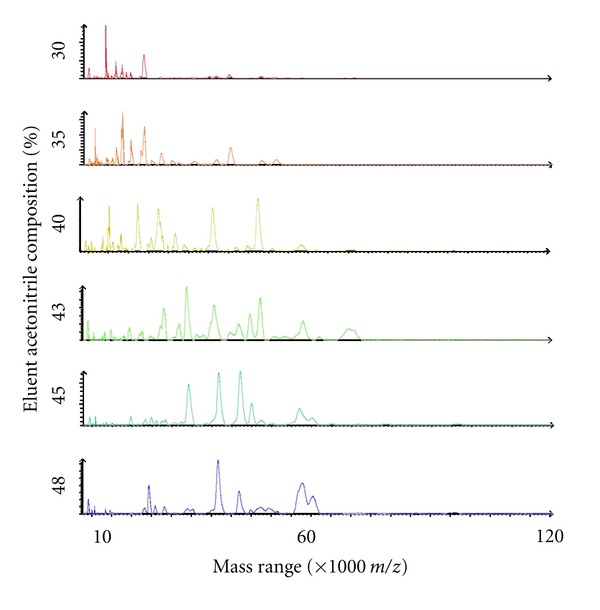
Exemplary linear-MALDI-TOF mass spectra of proteins in several different reversed-phase SPE fractions of a 0.25 M potassium chloride eluted anion-exchange fraction, derived from a DDF cytosolic extract of cultured human Neuro-2A cells. Proteins in fractions were directly cospotted with sinapic acid matrix and it is evident that such analysis is directly compatible with the MDLC fractionation protocol described herein.

**Figure 8:**
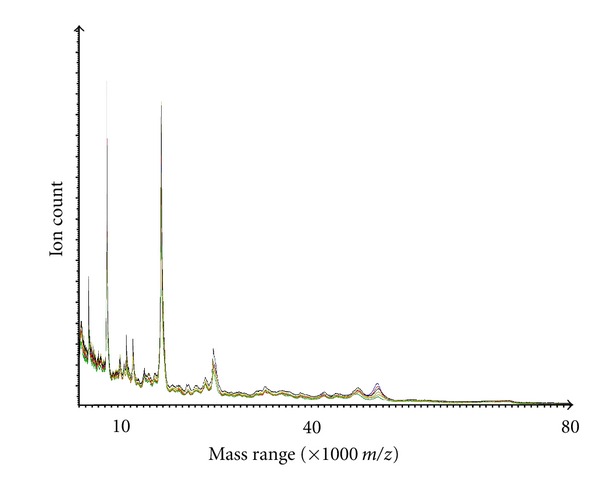
Exemplary comparative linear-MALDI-TOF mass spectra of proteins in 40% (v/v) acetonitrile eluted reversed-phase SPE fractions of 0.4 M potassium chloride eluted anion-exchange fraction, derived from DDF cytosolic extracts of six samples of cultured human Neuro-2A cells. Proteins in fractions were directly cospotted with alpha-cyano-4-hydroxy-cinnamic acid matrix. This data serves to show the high between sample extract precision with which some proteins were recovered and could be analysed.

As proof-of-concept, selected MDLC fractions derived from the cultured cells were profiled using mass-spectrometric approaches, as indicated from the linear mass spectra shown in Figure 7. Clearly, reversed-phase purified proteins are entirely amenable to analysis using MALDI-TOF-MS and whilst this has not thus far been routinely applied by us in our workflows, it does suggest that relatively low-cost, rapid, and high-throughput analyses could be achievable if desired. The high between sample extract precision with which some proteins were recovered and could be analysed is shown for six test samples derived from different cell samples (Figure 8). These results confirm the potential suitability of the described workflow for application to discovery profiling proteomic projects such as disease biomarker investigations. Similarly reversed-phase-SPE purified intact proteins have been readily analysed by us using Electrospray-TOF-MS demonstrating further applicability (data not shown).

### 3.5. Protein Quantitation Using Liquid-Chromatography-Tandem-Mass-Spectrometric Analysis

**Figure 9:**
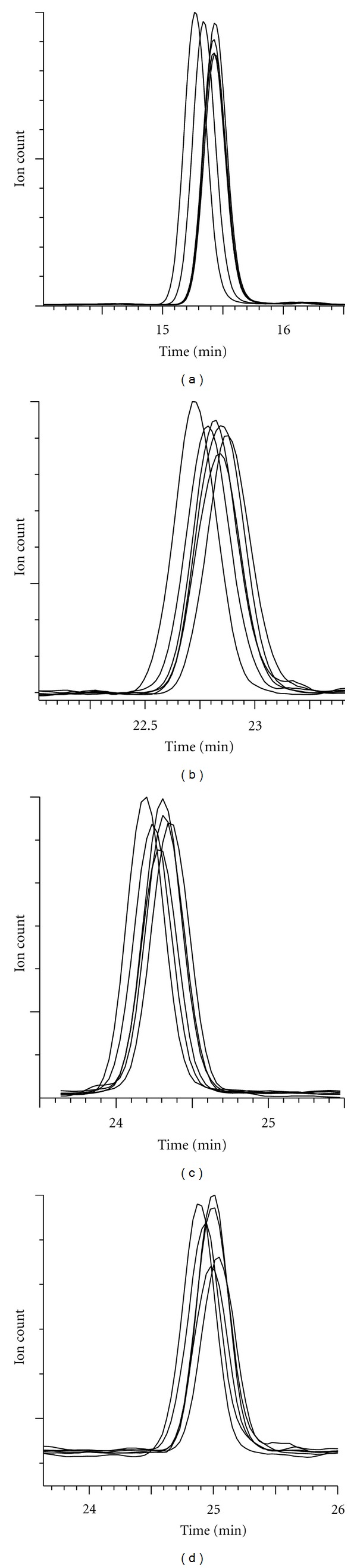
Exemplary LC-MS/MS (MRM) chromatogram peptide peaks from analyses of four candidate proteins in 35% (v/v) acetonitrile eluted reversed-phase SPE fractions of 0.15 M potassium chloride eluted anion-exchange fractions, derived from DDF cytosolic extracts of six samples of cultured human Neuro-2A cells. Target protein trypsinolytic signature peptides and the employed MRM transitions were (a) Ubiquitin [TITLEVEPSDTIENVK+2H]^2+^; 894.8 > 1002.5, (b) 60 s ribosomal subunit L30 [VCTLAIIDPGDSDIIR+2H]^2+^; 879.8 > 872.4, (c) serine-arginine-rich splicing factor 3 [NPPGFAFVEFEDPR+2H]^2+^; 811.8 > 1410.7, and (d) PML protein [NMSERSAMAAVLAMR+2H]^2+^; 827.3 > 933.5. This data serves to show the high between sample extract precision with which some proteins were recovered and confirms the potential suitability of the described workflow for application to quantitative proteomic projects.

Overlaid peptide MRM chromatogram peaks, measured for four proteins identified in 35% (v/v) acetonitrile eluted reversed-phase SPE fractions of 0.15 M potassium chloride eluted anion-exchange fractions, show similar peak properties (Figure 9). Note that differences in peak areas, for different test samples, indicate peptide (protein) abundance differences and so the relative quantification of specific proteins in the purified fractions is possible. This data serves to show the high between sample extract precision with which some proteins were recovered and confirms the potential suitability of the described workflow for application to quantitative proteomic projects subsequent to either direct proteolysis of MDLC fractions or to regions of SDS-PAGE profiled fractions (GeLC-MS) with subsequent LC-MS/MS.

### 3.6. Profiling Chromatographic Fractions Using 2-Dimensional Gel Electrophoresis

**Figure 10:**
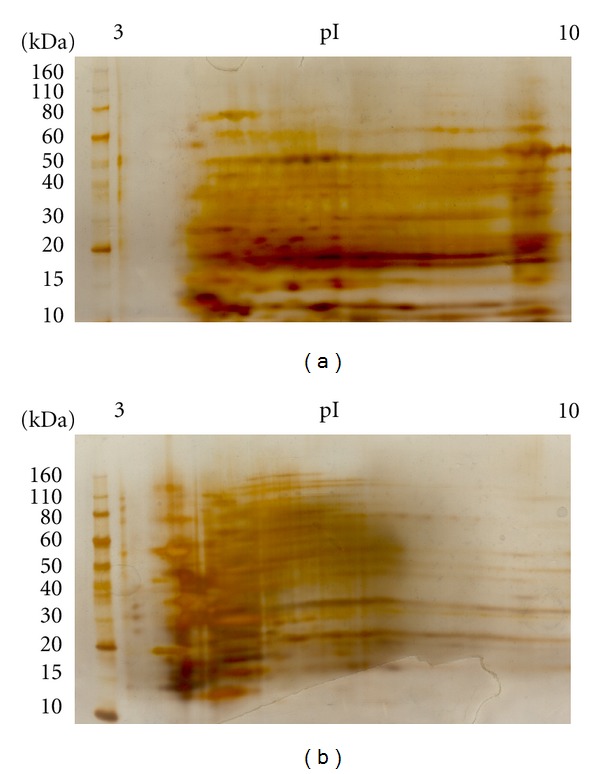
Representative examples of 2DE analysis (silver-stained) of two MDLC fractions. These are in both cases the 45% (v/v) acetonitrile reversed-phase SPE fractions of 0.25 M (a) and 0.4 M (b) potassium chloride eluted anion-exchange fractions, derived from a cytosolic DDF extract of cultured human Neuro-2A cells.

Whilst the multidimensional fractionation procedures described herein were developed to circumvent the application of classical 2DE and to achieve parallel and robust arraying of extracts, it was of interest to assess the compatibility of these protocols with 2DE. Figures 10(a) and 10(b) show exemplary 2DE gel profiles of two MDLC fractions and serve to highlight that less heterogeneous proteome fractions are obtainable using the described protocols and that fractions from this workflow are entirely compatible with 2DE. Differences in component pI in the two anion-exchange fractions are apparent, the average pI being lower in the 0.4 M anion-exchange fraction and this clearly demonstrates the resolving power of the protocol. Common anomalies associated with 2DE are apparent in the images of these gels further highlight the need for the development of new multidimensional protein separation approaches as described herein.

## 4. Discussion

In summary, we present an economical and high-throughput protocol for multidimensional fractionation of proteins, in this case extracted cytosolic proteins, which is compatible with various workflows and applicable to a range of experimental approaches. Initial fractionation using the DDF protocol yields four fractions, which (as demonstrated for the cytosolic fraction) can be further fractionated by anion-exchange chromatography. An additional dimension of separation is readily achieved by reversed-phase SPE separation of anion-exchange fractions, a method which we also demonstrate in its own right can be used to concentrate, desalt, and partially fractionate other complex proteome samples (shown for plasma, urine, and bacterial cell lysates). Where required, reversed-phase SPE fractions, in this case derived from anion-exchange fractions, can also be profiled by MALDI-TOF-MS or 2DE, offering an additional 1- or 2-dimensional separation, respectively and may also be used in quantitative proteomic projects where proteolytic peptides can be analysed using LC-MS techniques. Thus, precise 3-, 4-, or 5-dimensional protein separations can be easily achieved, largely without the requirement for specialist equipment, with the precision that should also allow comparative proteome analyses to be performed.
